# A cost-benefit analysis on the specialization in departments of obstetrics and gynecology in Japan

**DOI:** 10.1186/2191-1991-2-2

**Published:** 2012-03-27

**Authors:** Junyi Shen, On Fukui, Hiroyuki Hashimoto, Takako Nakashima, Tadashi Kimura, Kenichiro Morishige, Tatsuyoshi Saijo

**Affiliations:** 1 Faculty of International Studies, Hiroshima City University, 3-4-1 Ozuka-Higashi, Asa-Minami-Ku, Hiroshima 7313194, Japan; 2 Department of Obstetrics and Gynecology, Izumisano City Hospital, 2-23, Rinku Ourai Kita, Izumisanoshi, Osaka 5988577, Japan; 3 Department of Obstetrics and Gynecology, Kaizuka City Hospital, 3-10-20, Hori, Kaizukashi, Osaka 5970015, Japan; 4 Faculty of Service Industries, University of Marketing and Distribution Sciences, 3-1, Gakuen-Nishimachi, Nishi-ku, Kobe, Hyogo 651-2188, Japan; 5 Graduate School of Medicine, Osaka University, 2-2, Yamadaoka, Suita, Osaka 5650871, Japan; 6 Graduate School of Medicine, Gifu University, 1-1, Yanagido, Gifu City 501-1193, Japan; 7 Institute of Social and Economic Research, Osaka University, 6-1, Mihogaoka, Ibaraki, Osaka 5670047, Japan

**Keywords:** Cost-benefit analysis, Specialization, Departments of obstetrics and gynecology, Sensitivity analysis, Benefit-cost ratio

## Abstract

In April 2008, the specialization in departments of obstetrics and gynecology was conducted in Sennan area of Osaka prefecture in Japan, which aims at solving the problems of regional provision of obstetrical service. Under this specialization, the departments of obstetrics and gynecology in two city hospitals were combined as one medical center, whilst one hospital is in charge of the department of gynecology and the other one operates the department of obstetrics. In this paper, we implement a cost-benefit analysis to evaluate the validity of this specialization. The benefit-cost ratio is estimated at 1.367 under a basic scenario, indicating that the specialization can generate a net benefit. In addition, with a consideration of different kinds of uncertainty in the future, a number of sensitivity analyses are conducted. The results of these sensitivity analyses suggest that the specialization is valid in the sense that all the estimated benefit-cost ratios are above 1.0 in any case.

## Background

In April 2008, the specialization in departments of obstetrics and gynecology (OBGY) was conducted in Sennan area of Osaka prefecture in Japan, which aims at solving the problems of regional provisions of obstetrics service (e.g., shortage of obstetricians, over working of obstetricians, etc.).^a ^Under this specialization, the departments of OBGY in two city hospitals (i.e., Kaizuka City Hospital and Izumisano City Hospital) were combined as one medical center (i.e., the Mother and Child Medical Center), whilst Kaizuka City Hospital is in charge of the department of gynecology and Izumisano City Hospital operates the department of obstetrics.^b^

Several related previous studies on the provision of obstetrics services are found in the literature. [1], examined the relationships among competition, cost, and quality within the singular product and geographic market of obstetrics services at hospitals within the state of Missouri. His results indicated that increased competition is related to both increases in quality of care and costs-the characteristics of a price-insensitive market. This result has obvious implications on health policy debates focusing on enhancing market competition as an avenue for health care reform. [2], examined the impact on the hospitals and the communities if one hospital in each area closed obstetrics services due to excess capacity in each community. The main finding of this study is that five of the seven examined hospitals would be in better financial condition if they closed obstetrics and system-wide cost savings of 7-15% of the cost of obstetrics were computed if one unit per community were closed. Similarly, in a recent study, [3], analyzed the effects of hospital closures on social welfare and the local economy and found that the cost savings from closures of the studied hospitals are more than offset the reduction in patient welfare. However, a further finding in [3] indicates that because some of the cost savings are shared nationally, total surplus in the local community may decline following a hospital closure. Since the specialization analyzed in the current paper does not close any department in any hospital, it is possible to attain a total surplus in the local community.

The prime purpose of this paper is to evaluate whether the above mentioned specialization in departments of OBGY can be regarded as a valid approach improving the regional provision of obstetrical service in Sennan area. The dominant approach used in policy or project evaluation is cost-benefit analysis (CBA). CBA is the systematic and analytical process of comparing benefits and costs in evaluating the desirability of a policy or project. CBA is fundamental to government decision making and is established as a formal technique for making informed decisions on the use of society's scarce resources. It attempts to answer such questions as whether a proposed project is worthwhile, the optimal scale of a proposed project and the relevant constraints [4]. This raises the question that how to calculate social benefits (i.e., the sum of consumers' benefits and producers' benefits) appropriately. Comparing to producers' benefits that are ordinarily calculated by their revenues, consumers' benefits are much more difficult to be estimated properly. Traditionally, many researchers relied on the revealed preference (RP) approach (i.e., using market data) to estimate consumers' benefits via the *change in consumer surplus*. However, due to the difficulty of obtaining consumers' market data, much attention has been paid to the stated preference (SP) approach in recent years, which involves choice responses from hypothetical markets.^c ^Contingent valuation method (CVM) and choice experiment (CE) are two typical examples of the SP approach.

In this study, we conducted a hypothetical choice experiment survey to obtain the data for estimating consumers' benefits of pregnant women in Sennan area. The survey was conducted between March 10th and 12th in 2008. We posted the questionnaire to 2637 subjects who delivered their children in Kaizuka City Hospital during 2003 to 2007. 1081 valid responses were returned, with a satisfied collection rate at about 41%. In addition, producers' benefits and the associated costs generated from the specialization were calculated based on the data provided by Izumisano City Hospital.

The remainder of the paper is organized as follows. Section 2 describes in detail the specialization of OBGY departments in Sennan area of Osaka prefecture. Section 3 presents the methodology issues, which includes the model specification for estimating consumers' surplus via choice experiment method, the explanation of the choice experiment design, and the assumptions of the basic scenario for the CBA. Results and discussions are given in Sections 4 and 5, respectively. Finally, Section 6 draws the conclusion.

### The specialization in departments of OBGY in the Sennan area

**Figure 1:**
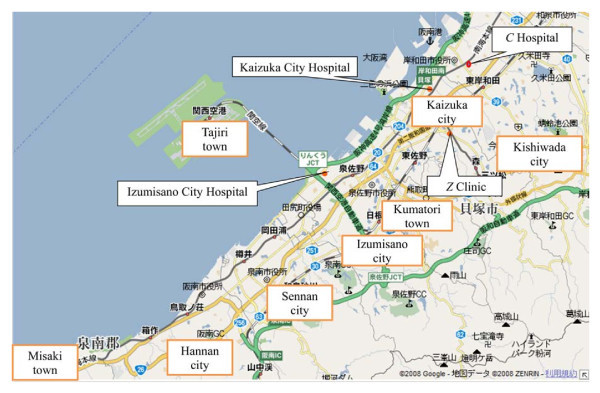
**Map of Sennan area**.

Sennan area includes five cities (Kishiwada, Kaizuka, Izumisano, Sennan, and Hannan) and three towns (Kumatori, Tajiri, and Misaki). Figure 1 shows the location of each city/town and the four maternity institutions used in the CBA. Table 1 provides the population and number of maternity institutions before the specialization in each city/town based on the statistics of Japanese Ministry of Internal Affairs and Communications [5]. From the table, we may calculate that in Sennan area the number of population per maternity institution is about 65 thousands in average.

**Table 1:** Population and number of maternity institutions in the Sennan area

	Population (thousands)	Number of maternity institutions	
		
		Hospital	Clinic
Kishiwada City	200	2	1
Kaizuka City	90	1	1
Izumisano City	100	2	0
Sennan City	65	0	0
Hannan City	58	0	2
Kumatori Town	44	0	0
Tajiri Town	8	0	0
Misaki Town	18	0	0

Notes: The figures of maternity institutions are those before the specialization. After the specialization, the number of maternity institutions in Kaizuka City became 0 for hospital and 1 for clinic (i.e., *Z *clinic)

Before the specialization, the number of obstetrician and gynecologist was 5 in Izumisano City Hospital and 5 in Kaizuka City Hospital, respectively. As each hospital carried out about 750 births per year, it is to say that in these two hospitals annual births per physician were about 150. According to the study of [6], this number is above the average births per physician in Japan (137.9), and above the average in Germany (143.1), USA (140.9), Denmark (126.2), Singapore (120.3), Finland (96.4), and Korea (95.9). Obviously, the obstetricians and gynecologists in these two hospitals bore a heavy burden.

In order to solve this problem, departments of obstetrics and gynecology in Kaizuka City Hospital and Izumisano City Hospital were integrated as the Mother and Child Medical Center in Sensyuu Area in April 2008. The center divides into two sub-centers. One is called Prenatal Care Center, which is operated by Izumisano City Hospital due to the reason that Izumisano City Hospital has the NICU (Neonatal Intensive Care Unit) and is nearer to the Accident and Emergency Center. Another sub-center is called Gynecological Care Center, which is operated by Kaizuka City Hospital. Both hospitals accept walk-in patients and conduct gynecological checkup and puerperal checkup. However, because Izumisano City Hospital specializes in the delivery and emergency service, the pregnant women examined in Kaizuka City Hospital will be moved to Izumisano City Hospital when risk of their birth is realized or they start having contractions. On the other hand, Kaizuka City Hospital specializes in gynecology, conducting scheduled surgeries and fertility treatments.

This specialization in departments of OBGY has three main merits. The first merit is that each hospital can concentrate in its specialized areas by fully utilizing its existed facilities. The second advantage is that it enables the provision of more advanced medical services as the specialized areas of each hospital are further enriched by this measure. Third, since the doctors from Kaizuka City Hospital are also involved in after-regular-hour-on-duty in Izumisano City Hospital, the overworking problem of obstetrician and gynecologist in both hospitals will be mitigated, to some extent, by the possible adoption of two-doctor-on-duty system.^d ^In Japan, because of the shortage of obstetricians and gynecologists, specializations among two hospitals such as closing the department of OBGY in one hospital and leaving that in another hospital are often implemented. However, different from these existed specializations, the specialization in Sennan area can be regarded as a new attempt in Japan as it would prevent the closure of any OBGY department in either hospital. If this specialization can be proved as a valid approach to improve the regional provision of obstetrical service, its application into other areas or other fields is expected.

Finally, there is another issue related to the specialization should be mentioned. Among 5 cities and 3 towns in Sennan area, only Kishiwada City does not participate in this specialization program. This is because each participating city/town must bear proportionate fees for any of their citizens using the services of Mother and Child Medical Center, and the Kishiwada City, as the most populous among them, would have to bear much higher fees than others. This issue may be considered as a possible factor influencing maternity institution choice among pregnant women in Sennan area after the specialization.

## Methods

### Choice model specification

Choice Experiment (CE) model is based on random utility theory. The basic assumption embodied in the random utility approach to choice modeling is that decision makers are utility maximizers, i.e. given a set of alternatives the decision maker will choose the alternative that maximizes his/her utility. The utility of an alternative for an individual (*U*) cannot be observed, however, it could be assumed to consist of a deterministic component (*V*) and a random error term (*ε*). Formally, individual *q*'s utility of alternative *i *can be expresses as:

(1) $$U_{iq}=V_{iq}+\varepsilon_{iq}$$

Hence the probability that individual *q *chooses alternative *i *from a particular set *J *that comprises *j *alternatives can be written as:

(2) $$P_{iq}=P\left(U_{iq}>U_{jq};\forall \;j\left(\neq i\right)\in J\right)=P\left(\varepsilon_{jq}<\varepsilon_{iq}+V_{iq}-V_{jq};\forall j\left(\neq i\right)\in J\right)$$

To transform the random utility model into a choice model, certain assumption about the joint distribution of the vector of random error terms is required. If the random error terms are assumed to follow the type I extreme value (EV1) distribution and be independently and identically distributed (IID) across alternatives and cases (or observations), the conditional logit model is obtained. In the conditional logit model, the choice probability in Equation (2) is expressed as:

(3) $$P_{iq}=\exp\left(V_{iq}\right)/\overset{J}{\underset{j=1}{\sum }}\exp\left(V_{jq}\right)$$

Then, making further assumption for the deterministic component of utility to be linear and additive in parameters, *V_iq _*= *β' X_iq_*, the probability in Equation (3) can be given as:

(4) $$P_{iq}=\exp\left(\beta^{\prime }X_{iq}\right)/\overset{J}{\underset{j=1}{\sum }}\exp\left(\beta^{\prime }X_{jq}\right)$$

where *X_iq _*is explanatory variable matrix of *V_iq _*and *β' *is the parameter vector associated with the matrix *X_iq_*. Following [7] and [8], the expected change in consumer surplus of individual *q *can be computed as:

(5) $$\Delta CS=\frac{1}{\lambda }\left[\ln\underset{j\in J}{\sum }\exp\left(V_{jq}^{2}\right)-\ln\underset{j\in J}{\sum }\exp\left(V_{jq}^{1}\right)\right]$$

where *λ *is the marginal utility of income, i.e. the estimated parameter of cost or price variable. *V*^2 ^and *V*^1 ^are the mean of the indirect utility for scenarios after and before implementing the evaluated policy, respectively. Finally, the total change in consumer surplus can be obtained by multiplying the amount obtained from Equation (5) with the number of people influenced by the evaluated policy and the probability estimated from Equation (4).

Summarizing the above descriptions, there are three steps to obtain the total change in consumer surplus. The first step is to estimate the parameters (i.e., the marginal utility) of each attribute in the choice model by conditional logit specification. The second step is to calculate the change in consumer surplus of the representative individual by applying Equation (5). Note that the purpose of Equation (5) is to transfer the utility into monetary benefits. Finally, the third step is to obtain the total change in consumer surplus by multiplying the result from the second step with the number of people and the probability estimated from Equation (4).

### Choice experiment design

In the choice experiment, three unlabeled alternatives (maternity institution A, maternity institution B, and maternity institution C) were provided. A number of attributes and assigned levels as indicated below were designed to generate hypothetical scenarios. Each alternative has seven common attributes.

➢ Cost of a birth: 300,000 JP¥, 360,000 JP¥, 420,000 JP¥, 480,000 JP¥

➢ Traveling time (by car) to the maternity institution: 5 minutes, 15 minutes, 25 minutes, 35 minutes

➢ Waiting time for a medical exam: 30 minutes, 60 minutes, 90 minutes, 120 minutes

➢ Medical exam in early-evening and weekends: Yes, No

➢ Number of obstetrician and gynecologist: 2, 4, 6, 8

➢ Number of nursing staff: 5, 10, 15, 20

➢ Pediatricians on duty: None, Only in the daytime, 24 hours

**Figure 2:**
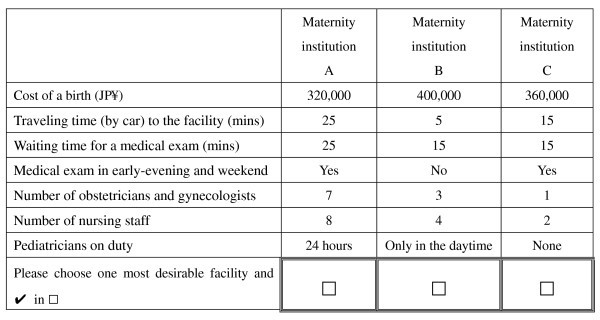
**An example of choice sets**.

Choice experiment design is to create the choice sets in an efficiency way, i.e., how to combine attributes and attribute levels into the profiles of alternatives and how to combine profiles into choice sets [8]. If we applied a full factorial design, it would generate too many choice sets that would be cumbersome to respondents to answer, even we divided these choice sets into a number of versions in questionnaire. In this study, we adopted the D-optimal design approach for choice experiments based on multinomial logit model. The objective of the D-optimal design is to extract the maximum amount of information from the respondents subject to the number of attributes, attribute levels and other characteristics.^e ^As a result of running the D-optimal design through Design-Expert 7.0 (Stat-Ease, Inc.), we created 22 choice sets. These choice sets were further randomly divided into 2 versions and randomly sent one version to the respondents, i.e., each version of the questionnaire consisted of 11 choice sets and each respondent answered 11 choice sets. One example of choice sets is presented in Figure 2.

### Sample description

The summary of demographic characteristics of our sample is provided in Table 2. From the summary, we found that 440 respondents (40.7% of the sample) were aged 30 to 34 and 727 respondents (67.3% of the sample) were full-time housewives. A majority of the respondents (82.7%) had one or two children. Similarly, 71.2% of the households consisted of three or four persons. In addition, the distribution of educational level and annual household income revealed that 42.9% of the sample had undergraduate degree and 65.5% of the sample had annual household income between 2 and 6 million JP¥ (about 25,000 to 75,000 US$ if 1 US$ = 80 JP¥).^f^

**Table 2:** Socio-demographic characteristics of the sample

Characteristics	n	%	Characteristics	n	%
*Age (years) *			*Household size *		
Below 20	8	0.7	1 person	1	0.1
20 - 24	72	6.7	2 persons	9	0.8
25 - 29	173	16.0	3 persons	338	31.3
30 - 34	440	40.7	4 persons	431	39.9
35 - 39	297	27.5	5 persons	184	17.0
Over 40	88	8.1	Above 6 persons	113	10.5
No answer	3	0.3	No answer	5	0.5
*Educational level *			*Annual household income *		
Senior high school	374	34.6	Below 2 million JP¥	43	4.0
Technical degree	183	16.9	2 - 3.999 million JP¥	328	30.3
Undergraduate degree	464	42.9	4 - 5.999 million JP¥	380	35.2
Master degree	8	0.7	6 - 7.999 million JP¥	135	12.5
Others	24	2.2	Above 8 million JP¥	86	8.0
No answer	28	2.6	No answer	109	10.1
*Occupation *			*Number of children *		
Full-time employed	187	17.3	1	394	36.6
Part-time employed	159	14.7	2	497	46.1
Student	4	0.4	3	158	14.7
Full-time housewife	727	67.3	Above 4	28	2.6
No answer	4	0.4	No answer	4	0.37
Total observations	1081	100	Total observations	1081	100

### Basic scenario

The following items of benefit and cost are considered in the current study to evaluate the validity of the specialization in departments of OBGY.

Benefit items

➢ The change in consumer surplus of pregnant women in Kaizuka City, Izumisano City, Hannan City, and Sennan City^g^

➢ The change in producer surplus of Izumisano City Hospital

Cost items

➢ Extra investment in equipment

➢ Extra personnel cost

➢ Extra running cost

In order to estimate the change in consumer surplus, a number of issues should be solved in advance. First, the basic scenarios of all the attributes used in the choice experiment before and after the specialization for the associated maternity institutions should be assumed. We provide these scenarios for Izumisano City Hospital, Kaizuka City Hospital, *Z *Clinic, and *C *Hospital in Table 3.^h ^It should be noted that all these scenarios are based on the actual status of each institution before and after the specialization. Next, concerning the future number of pregnant women in Kaizuka City, Izumisano City, Hannan City, and Sennan City, we assume that under the basic scenario the pregnant women growth rate in these cities is zero. In other words, we assume that the number of pregnant women in the future is the same as that in the evaluation point (i.e., the year of 2008).^i ^In addition, the change in producer surplus (i.e., the extra revenue of obstetrics department due to the specialization) and extra costs are calculated based on the data provided by Izumisano City Hospital. The change in producer surplus in the evaluation point is calculated by subtracting the sum of revenues of obstetrics departments in Kaizuka City Hospital and Izumisano City Hospital in 2007 from the revenue of OBGY department in Izumisano City Hospital in 2008, while extra costs are calculated as the sum of extra investment in equipment, extra personnel cost and extra running cost. Note that the change in producer surplus in the future and extra personnel and running costs are assumed to be same as those in 2008, while the extra investment in equipment is assumed to occur only in 2008. Finally, the evaluation criteria under the basic scenario (e.g., social discount rate, evaluation period) are provided in Table 4. These criteria are normally applied in other cost benefit analyses in Japan.

**Table 3:** Basic scenarios of the associated maternity institutions

	Izumisano Hospital	Kaizuka Hospital	*Z *Clinic	*C *Hospital
*Cost of a birth (10,000JP*¥*)*				
Izumisano citizens	35 (35)	33.5 (-)	45(45)	43.5(43.5)
Kaizuka/Hannan/Sennan citizens	40(35)	33.5(-)	45(45)	43.5(43.5)

*Traveling time by car(mins) *^a^				
Kaizuka citizens	18(18)	17(-)	13(13)	13(13)
Izumisano citizens	13(13)	15(-)	13(13)	20(20)
Hannan citizens	23(23)	36(-)	33(33)	41(41)
Sennan citizens	15(15)	27(-)	23(23)	33(33)

*Waiting time for an exam(mins) *	120(90)	120(-)	90(90)	120(120)

*Early-evening/weekend exams *^b^	0(0)	1(-)	1(1)	1(1)

*Obstetricians and gynecologists *	6(13)	5(-)	1(2)	2(2)

*Nursing staff *	15(19)	12(-)	8(8)	10(10)

*Pediatricians on duty(hours) *	9(9)	9(-)	0(0)	3(3)

Notes: Scenarios before the specialization are listed without parentheses, while scenarios after the specialization are listed in the parentheses. ^a ^Traveling time by car to the facility is a mean value of each respondent's traveling time from her residence to the associated facility. ^b ^0 denotes that the facility does not provide early-evening exam and weekend exam, while 1 denotes that the facility has early-evening exam and weekend exam

**Table 4:** Evaluation criteria under the basic scenario

Social discount rate	Evaluation period	Evaluation point	Indicator
4.0%	40 years	2008	Benefit-cost ratio(B/C

## Results

Table 5 lists the conditional logit model estimation results and Table 6 presents the cost benefit analysis results under the basic scenario. As shown in Table 5, all the estimated parameters are statistically significant with the expect signs in all sub-samples. Based on these parameters and the basic scenarios presented in Table 4, the change in consumer surplus for the representative pregnant woman in Izumisano City, Kaizuka City, Hannan City, and Sennan City can be obtained according to Equation (5), respectively.^j ^After multiplying these individual amounts with the number of pregnant women in each city and the corresponding choice probability of the Izumisano City Hospital in each city, we obtain changes in consumer surplus of pregnant women in each city in the evaluation point. Then, by summing up these figures and applying the evaluation criteria in Table 4, the present value of change in consumer surplus is estimated at 1.763 billion JP¥. On the other hand, based on the calculation methods presented in Table 6, the present values of the change in producer surplus and extra costs are estimated at 1.994 billion JP¥ and 2.748 billion JP¥, respectively. The benefit-cost (B/C) ratio 1.367 indicates that due to being able to generate 36.7% or 1.009 billion JP¥ net benefit, the specialization in departments of OBGY in Sennan area can be regarded as a success.

**Table 5:** Estimation results by the conditional logit model

	Kaizuka citizens	Izumisano citizens	Hannan citizens	Sennan citizens
Cost of a birth	-0.140**	-0.121**	-0.200**	-0.168**
Traveling time (by car) to the facility	-0.034**	-0.027**	-0.010*	-0.011*
Waiting time for a medical exam	-0.012**	-0.013*	-0.010**	-0.019*
Early-evening and weekend exams	0.894**	0.991**	1.037**	1.483**
Number of obstetricians and gynecologists	0.158**	0.173**	0.343*	0.275^cph^
Number of nursing staff	0.075**	0.078**	0.072**	0.077*
Pediatricians on duty	0.074**	0.077**	0.092*	0.074*

Notes: * and ** denote the parameter is significantly different from zero at 5% and 1% level, respectively. The estimated parameter of "cost of a birth" variable is the marginal utility of income

**Table 6:** Benefits and costs under the basic scenario

*Benefits items (the unit of the figures is billion JP¥)*	
(1) change in consumer surplus of pregnant women in Kaizuka city in 2008	0.020
(2) change in consumer surplus of pregnant women in Izumisano city in 2008	0.025
(3) change in consumer surplus of pregnant women in Hannan city in 2008	0.022
(4)change in consumer surplus of pregnant women in Sennan city in 2008	0.022
(5) total change in consumer surplus in 2008 (=(1) + (2) + (3) + (4))	0.089
**(6) present value of change in consumer surplus**	**1.763**
(7) revenue of OBGY department in Izumisano City Hospital in 2008	0.850
(8) revenue of obstetrics department in Izumisano City Hospital in 2007	0.321
(9) revenue of obstetrics department in Kaizuka City Hospital in 2007	0.431
(10) total change in producer surplus in 2008 (=(7) - (8) - (9))	0.098
**(11) present value of change in producer surplus**	**1.994**
**(12) present value of benefits (=(6) + (11))**	**3.757**

***Cost items (the unit of the figures is billion JP¥)***	
(13) extra investment in equipments in 2008^a^	0.227
(14) extra personnel cost in 2008	0.125
(15) extra running cost in 2008	0.010
**(16) present value of extra costs**	**2.748**

***Benefit-cost (B/C) ratio (=(12)/(16))***	**1.367**

Notes: The year of 2008 is the evaluation point. All the present values are calculated based on the 4.0% discount rate and the period of 40 years. ^a ^Extra investment in equipment is assumed to occur only in 2008

The estimated B/C ratio under the basic scenario is plausible. However, as we know, there exist many kinds of uncertainty in the future when applying the CBA approach. Such uncertainty will surely lead to different B/C ratios and in turn different policy implications. To solve this problem, a sensitivity analysis is normally applied in the CBA approach. Usually, it is done by first making some adjustments in a reasonable range for the basic scenario and then recalculating the corresponding B/C ratios. With these B/C ratios in hand, policy decision makers and/or those involved in the evaluated policy are able to make their judgments upon considering various uncertainties besides the basic scenario.

In the current study, we first conducted the sensitivity analyses by adjusting the social discount rate and evaluation period. The results are presented in Table 7. As shown in the table, for the range of the social discount rate from 1% to 7% and the evaluation period from 30 years to 50 years, all the corresponding B/C ratios are above 1.0, which suggests that the uncertainties of these two items do not affect the results obtained under the basic scenario.

**Table 7:** Sensitivity analysis for changing the social discount rate and evaluation period

Social discount rate	1.0%	2.0%	3.0%	4.0%	5.0%	6.0%	7.0%
B/C ratio (30 years)	1.368	1.368	1.367	1.367	1.366	1.366	1.365
B/C ratio (40 years)	1.369	1.368	1.368	1.367	1.367	1.366	1.366
B/C ratio (50 years)	1.369	1.369	1.368	1.368	1.367	1.366	1.366

Second, the sensitivity analysis results for adjusting the operation patterns of Izumisano City Hospital as shown in Table 8 also provide the evidence that this specialization in departments of OBGY is successful. Especially, all the B/C ratios of the patterns, such as reducing the waiting time for an exam to 60 minutes, providing 24-hour schedule of pediatricians, and offering early-evening and weekend exams are all above 1.5, implying that there is still a room to further promote regional social welfare as a whole. Finally, concerning the sensitivity analysis for changing the number of future pregnant women, we considered two scenarios, i.e., pessimistic scenario and extremely pessimistic scenario. As shown in Table 9, the B/C ratio is above 1.0 in either case, which confirms that the specialization in departments of OBGY is still a plausible approach even with the growing problem of low birthrate in Sennan area.

**Table 8:** Sensitivity analysis for changing the operation patterns of the Izumisano City Hospital

	lower scenario	base scenario	upper scenario
Waiting time for a medical exam (mins)	60	90	120
B/C ratio	1.639	1.367	1.114

Number of obstetricians and gynecologists^a^	10	13	16
B/C ratio	1.254	1.367	1.461

Number of nursing staff^b^	15	19	23
B/C ratio	1.170	1.367	1.357

Pediatricians on duty^c^	Only half day	Only in the daytime	24 hours
B/C ratio	1.081	1.367	1.566

Early-evening and weekend exams^d^		No	Yes
B/C ratio		1.367	1.701

Notes:^a ^A decrease (increase) of 30 million JP¥ in extra personnel cost per year is posted in the analysis, corresponding to the situation of decreasing (increasing) 3 individuals relative to the current number of obstetricians and gynecologists (13 individuals). ^b ^A decrease (increase) of 21.36 million JP¥ in extra personnel cost per year is posted in the analysis, corresponding to the situation of decreasing (increasing) 4 individuals relative to the current number of nursing staff (19 individuals). ^c ^An increase of 50 million JP¥ in extra personnel cost per year is posted in the analysis, corresponding to the situation of changing the system of pediatricians on duty from the current status (only in the daytime) to 24 hours. ^d ^An increase of 30 million JP¥ in extra personnel cost per year is posted in the analysis, corresponding to the situation of starting early-evening and weekend exams

**Table 9:** Sensitivity analysis for changing the number of future pregnant women

Year	Pessimistic scenario				Extremely pessimistic scenario			
	
	Kaizuka	Izumisano	Hannan	Sennan	Kaizuka	Izumisano	Hannan	Sennan
2008 ~ 2017	0%	0%	0%	0%	-5%	-5%	-5%	-5%
2018 ~ 2027	-5%	-5%	-5%	-5%	-8%	-8%	-8%	-8%
2028 ~ 2037	-8%	-8%	-8%	-8%	-10%	-10%	-10%	-10%
2038 ~ 2047	-10%	-10%	-10%	-10%	-20%	-20%	-20%	-20%

B/C ratio		1.341			1.303			

Note: The influence of changing the number of future pregnant women on the change in producer surplus and extra costs is not considered

## Discussion

The above results exhibit that in any cases the total benefit is larger than the total cost after the specialization of OBGY departments in two city hospitals. However, we should be vividly aware that the net benefit of the specialization would not be generated if without considering the consumers' benefits. It is to say that when evaluating a project or policy related to the supply side, both consumers' and producers' benefits should be taken into account. In our case, the benefits of pregnant women were generated by the facts that after the specialization (i) cost of a birth was reduced; (ii) waiting time for an exam was reduced; and (iii) the number of doctors and nursing staffs increased in Izumisano City Hospital. In a detailed follow-up calculation under the basic scenario, we found that among the total consumer benefits, the monetary factor (i.e., the first factor) contributed about 49.6% (0.874 billion JP¥) and the non-monetary factors (i.e., the second and third factors) contributed about 50.4% (0.889 billion JP¥).^k ^Obviously, if we only took the monetary factor into account, as several previous studies did in their CBAs, the B/C ratio would be 1.024-very close to a rock-bottom ratio that can generate net benefits. Therefore, it is important for economists to take the non-monetary factors into account when applying a CBA.

Based on the results under basic scenario, the specialization in OBGY departments can generate 1.009 billion JP¥ net social benefits. Naturally, it yields another important question-which part actually benefits from these additional profits? Consumers or the hospital or both? To answer this question, we did another follow-up calculation. Based on the data provided by Izumisano City Hospital, we found that the additional costs of 2.748 billion JP¥ were financed by three parts (i) subsidy provided by Osaka Prefecture in 2008 (0.025 billion JP¥); (ii) contributions from the municipalities that participated in the specialization program (1.561 billion JP¥ in present value in 2008); and (iii) Izumisano City Hospital (1.162 billion JP¥ in present value in 2008). Therefore, among the 1.009 billion JP¥ net benefits, Izumisano City Hospital profits 82.5% or 0.832 billion JP¥ (= 1.994 - 1.162) and pregnant women in Sennan area enjoy 17.5% or 0.177 billion JP¥ (= 1.009 - 0.832). This result is exciting because the specialization brings not only net social benefits but also both consumer and producer sides benefit from these additional profits. It suggests that the specialization program is a win-win approach for both consumer and producer sides.

The success of the specialization in departments of OBGY has a two-fold significance. First, it does exhibit that the consumption and provision of obstetrical service in Sennan area beneficially change based on a welfare analysis from both consumer and producer sides. Second, as a possible means to solve the problem of obstetrical service provision, this specialization method may be worthy of application in other areas of Japan and even in other countries. However, it should be noted that just copying the specialization in Sennan area into other areas of Japan or other countries may have a risk considering the existence of regional differences in the provision of obstetrical and gynecological services is conceivable. Furthermore, the factors influencing maternity institution choice among pregnant women in other areas or countries may be also different from those in Sennan area. Therefore, policy decision makers should be cautious not to just "copy and paste" the measure. Instead, they will need to conduct a throughout pre-survey on site to determine the applicability of this specialization measure.

There is one issue to be noted. In the current study, the evaluation focuses on obstetrics side but not on gynecology side. We have not conducted a survey for gynecological patients. Although the effect of the specialization is generally considered more significant on the obstetrics side than gynecology side, for the sake of completeness, we need the analysis on gynecology side, which we shall conduct in the near future as a continuation of the current study.

## Conclusion

The current paper provides a cost-benefit analysis on evaluating whether the specialization in departments of OBGY in Sennan area of Osaka prefecture is successful. All the estimated B/C ratios either under the basic scenario or in the sensitivity analyses taking various uncertainties into consideration are above 1.0, implying that the specialization is quite acceptable in terms of generating social net benefit.

## Endnotes

^a^The shortage of the obstetrician and gynecologist workforce is generally seen not only in Japan, but also in other western countries such as the US [9], Australia [10], etc.

^b^A detailed description of this specialization is provided in Section 2.

^c^More detailed discussions on the advantages and disadvantages between the RP and SP approaches can be found in [11] and [12].

^d^In Japan, the two-doctor-on-duty system in OBGY departments is usually considered as a valid measure to respond to the emergency births out of regular hour. One referee asked how the specialization could solve the shortage of physicians in two hospitals' OBGY departments while the number of physicians did not change. In fact, the shortage of physicians in two hospitals' OBGY departments was mainly caused from the shortage of obstetrician for emergency births out of regular hour. In our view, this problem could be solved by having physicians from Kaizuka City Hospital be also involved in after-regular-hour-on-duty in Izumisano City Hospital because the distance between two hospitals (about 6.5 km) is not too long.

^e^For more details on the D-optimal design, see [8,13] and [14].

^f^One referee asked whether our sample was a representative sample. Due to the fact that there is no published data on the socio-demographic characteristics of pregnant women in the surveyed area, we are unable to carry out statistical tests on investigating the representativeness of our sample.

^g^Consumers' benefits of pregnant women in Kumatori Town, Tajiri Town, and Misaki Town should also be included. However, in our sample the number of respondents from these three towns is too few to ensure the robustness of the estimated parameters. Therefore, we only estimate consumers' benefits in Kaizuka City, Izumisano City, Hannan City, and Sennan City.

^h^*Z *clinic is the unique maternity institution in Kaizuka City after the specialization and *C *Hospital is the largest maternity institution in Kishiwada City.

^i^According to Japanese Ministry of Internal Affairs and Communications [15], Japan's total population reached its peak in 2004 and was stable between 2005 and 2010. Their prediction indicates that there will be a decreasing trend in the next 40 years, especially after 2030. For this reason, we also consider two scenarios about decreasing the number of future pregnant women in the sensitivity analysis.

^j^It should be noted that the estimated parameter of "cost of a birth" variable in Table 5 is indeed the marginal utility of income *λ *in Equation (5). Therefore, by applying Equation (5), the utility could be transferred into monetary benefits.

^k^The calculation was done by estimating the corresponding consumer surpluses when keeping only one factor varied and other factors constant.

## Competing interests

The authors declare that they have no competing interests.

## Authors' contributions

The authors carried out the analysis and wrote the paper together. All the authors read and approved the final manuscript.
